# Neuropsychiatric Manifestations in a Patient with Panhypopituitarism

**DOI:** 10.1155/2017/5082687

**Published:** 2017-05-08

**Authors:** Oluwole Jegede, Ajouka Jeyakumar, Thyarapan Balakumar, Alyssa Raghu, Katherine I. Chang, Katarina Soewono, Mario Gustave, Ayodeji Jolayemi

**Affiliations:** ^1^Interfaith Medical Center, Brooklyn, NY, USA; ^2^American University of Antigua, Osbourn, Saint Kitts and Nevis; ^3^Medical University of the Americas, Charlestown, Saint Kitts and Nevis

## Abstract

We present a case of an incidental diagnosis of panhypopituitarism in a 68-year-old African American man admitted to our psychiatric inpatient unit with symptoms suggestive of schizophrenia. The case was unusual as a first-episode psychosis given the patient's age. In the course of his admission, the patient's clinical condition deteriorated culminating in a sudden altered mental status which prompted a transfer to the medical floors and further investigations. A head CT scan and a pituitary MRI revealed a near total resection of the pituitary while laboratory investigations revealed hyponatremia and a grossly low hormone profile. The progression of these events casts doubts on our admitting diagnosis as the primary cause of the patient's symptoms. The patient's clinical condition improved only when his endocrinopathy was treated with hormone replacement, fluids, and electrolyte correction in addition to antipsychotics. An inability to verify the patient's psychiatric history and a remote history of pituitary resection several decades earlier, unknown to the treating team, added to the diagnostic conundrum. We revised the diagnosis to neuropsychiatric manifestations secondary to an organic brain syndrome due to a partial pituitary resection. The patient was discharged with no symptoms of psychosis, good insight, judgment, and good reality testing.

## 1. Case Presentation

The patient is a 68-year-old single, unemployed, homeless African American man who was brought in to the psychiatric emergency room by city emergency services following a reported agitation and aggressive behavior. The patient was unable to give a history of the circumstances of his admission. He appeared disheveled, with a grossly disorganized speech and behavior, his thought process was nonlinear, illogical, and lacked associational quality. His thought content was significant for delusions of paranoia, grandiosity, and rife with religious themes. He stated that he was God and that he possessed “special powers to cure all diseases”; he also complained that people were “talking about me and stalking me to steal my millions of dollars.” He endorses noncommand auditory hallucinations but denies formal thought disorders including thought insertion, thought withdrawal, or thought broadcasting. He denied suicidal or homicidal ideations. Initial routine laboratory investigations were within normal limits and urine toxicology was negative of illicit drugs. The patient was determined to be acutely psychotic, a danger to himself and others, and in need of inpatient stabilization. He was diagnosed with schizophrenia and started on antipsychotics and mood stabilizers.

## 2. Hospital Course

After three weeks on admission, his clinical condition appeared to be getting worse; he also developed sudden onset altered mental status with marked disorientation, waxing and waning consciousness, and worsening visual and auditory hallucinations. Further work-up including laboratory investigation revealed significant hyponatremia that may have contributed to the marked disorientation. The medical team was consulted and a transfer to the medical floor recommended.

### 2.1. Laboratory Investigations

The patient's admission Complete Blood Count (CBC), kidney, liver function tests, and urine toxicology were within normal limits. Serum sodium and potassium were 137 mmol/L (136–144.0 mmol/L) and 4.2 mmol/L (3.6–5.1), respectively. Other routine urine analyses and coagulation profiles were also within normal limits as were routine chest radiograph and ECG. On transfer to the medical floors, a complete hormonal profile was carried out with a clinical suspicion of hypopituitarism. Serum cortisol (AM) was 1.8 mg/dL (6.2–19.4 mg/dL). Serum thyroid-stimulating hormone and free T4 were 0.084 uIU/ml (0.450–4,500 uIU/ml) and 1.31 ng/ml (0.82–1.77 ng/dL), respectively. Serum testosterone level was 3 ng/dL (348–1197 ng/dL) and luteinizing hormone and follicle-stimulating hormone were 0.1 mIU/ml (1.7–8.6 mIU/ml) and 1.0 mIU/ml (1.5–12.4 mIU/ml). Basal serum IGF1 levels were 46 mg/mL (47–192 mg/mL), respectively. Overall the patient's pituitary hormone profile was suggestive of panhypopituitarism. At this point, repeat chemistries showed hyponatremia of 128 mmol/L (136–144 mmol/L).

### 2.2. Radiologic Investigations

**Figure 1:**
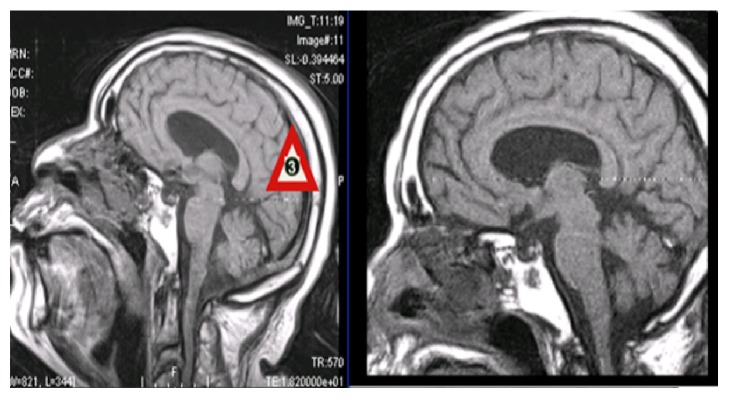
Sagittal T1 diffusion weighted MRI showing a complete opacification of the left sphenoid sinus.

A sagittal T1, axial T1, T2, diffusion-weighted and FLAIR, and coronal T1 multiplanar, multisequence MRI imaging of the brain without injection of gadolinium showed prominent ventricles and subarachnoid spaces suggestive of gross atrophy. There was also a complete opacification of the left sphenoid sinus. Multiplanar, multisequence imaging of the brain and pituitary gland without injection of gadolinium also suggested a transsphenoidal resection of the right lobe of the pituitary gland. A residual versus recurrent 8 mm soft tissue signal was identified along the lateral right sella turcica with a deviation of the stalk to the left as well as an opacification of the left sphenoid sinus as shown in Figure 1. The patient improved significantly over the next few weeks on the medical floors on antipsychotics, sodium replacement (sodium chloride tabs 1 g three times daily for hyponatremia), and hormonal replacement therapy including hydrocortisone 60 mg IVPB (intravenous) every six hours, levothyroxine 125 mcg daily, testosterone cypionate 200 mg IM (intramuscular) daily as well as a continuation of antipsychotic medication, and risperidone 2 mg PO (oral) twice daily. He was also placed on Keppra 500 mg PO (oral) daily for seizure prophylaxis. The patient's mental status improved significantly and was cleared medically to return to the psychiatric floors.

The patient was later able to give a history of a pituitary resection when he was 30 years old following a 6-month history of blurry vision and generalized weakness, presumably secondary to prolactinoma. Following the surgery, he reported that he only complied with about 6 months of postsurgical recommendation of hormone replacement. In the years following this event, the patient reported a twenty-year uninterrupted work history and good interpersonal relationships with adequate social and occupational functioning. We were able to verify his job history and places of domicile. His mental health history however remained sketchy and inadequate; he denied any diagnosis of mental illness or being on any antipsychotic medications prior to this hospitalization.

The patient's diagnosis was revised to a psychotic disorder due to an endocrinopathy secondary to a partial pituitary resection. The patient continued to improve clinically; at discharge the treating team determined that the patient was no longer a danger to himself or others and his thought process was goal directed, logical, linear, and with good associational quality. His thought content was devoid of delusions or hallucinations and he displayed an intact reality testing with a good judgment and insight.

## 3. Discussion

The pathophysiologic presentation of panhypopituitarism is well documented in literature but there appears to be a paucity of data on its possible associated neuropsychiatric manifestations [1, 2]. Cases described in literature involve psychotic presentations following panhypopituitarism from various etiologies including an ectopic posterior pituitary, Russell's Viper Bite, Sheehan's syndrome, Traumatic Brain Injury (TBI), and after glucocorticoid therapy [2–6]. Mechanisms for the pathogenesis of psychosis in hypopituitarism may be a result of interactions between pituitary hormones and the dominant neurotransmitters: serotonin, dopamine, GABA, and glutamate and a complex metabolic and electrolyte changes in the central nervous system resulting from a combination of hypothyroidism, hypoglycemia, and low cortisol. Krishnamurthy et al. (2013) in a study of molecular changes of post mortem pituitary glands from schizophrenia subjects described differentially expressed molecules of the hypothalamic-pituitary-adrenal axis such as cortisol, proadrenocorticotropic hormone, arginine vasopressin precursor, agouti-related protein, growth hormone, prolactin, and secretagogin, as well as molecules associated with lipid transport and metabolism, such as apolipoproteins A1, A2, C3, and H. They concluded that these hormones may present some diagnostic utility with further research [7].

Pariante et al., 2014, reported a 10% larger pituitary volume in patients with first episode psychosis suggestive of HPA axis hyperactivity and 17% smaller pituitary in patients with established schizophrenia possibly due to repeated episodes of HPA axis hyperactivity [8]. Thyroid hormones deregulation has also been reported as a common feature in schizophrenia; available evidence suggests that the pituitary-thyroid axis is involved in the serotonergic, dopaminergic, glutamatergic, and GABAergic networks, as well as myelination and inflammatory processes [9].

The etiology of psychosis in our patient is difficult to ascertain for two main reasons: one, our inability to obtain a comprehensive psychiatric and medical history (which he denies), and two, the relatively late onset of psychosis and his history of being symptom-free for so many years following the pituitary resection. These factors make it problematic to rule out an underlying psychotic disorder; however, it is of clinical significance that, once we added hormone supplementation to the patient's medication regimen, he made remarkable progress. As more reports emerge that indicate the neuropsychiatric manifestations of panhypopituitarism, perhaps a more comprehensive endocrine work-up may be considered in certain cases of psychosis of atypical presentations. The criteria for determining when to consider endocrine work-up needs to be further explored. The exploration of such criteria will make an interesting further research direction.
